# *Chordodes ferox*, a new record of horsehair worms (Nematomorpha, Gordiida) from South Africa

**DOI:** 10.3897/zookeys.566.6810

**Published:** 2016-02-18

**Authors:** Andreas Schmidt-Rhaesa, Renzo Perissinotto

**Affiliations:** 1 Zoological Museum, Centrum für Naturkunde, University Hamburg, Martin-Luther-King-Platz 3, 20146 Hamburg, Germany; 2 SARChI Chair in Shallow Water Ecosystems, Nelson Mandela Metropolitan University, PO Box 77000, Port Elizabeth 6031, South Africa

**Keywords:** Nematomorpha, Chordodes ferox, praying mantid hosts, new records, South Africa

## Abstract

Three females and one male specimen of a previously unconfirmed species of horsehair worms (Nematomorpha) from South Africa are described using Scanning Electron Microscopy. The females correspond to the description of *Chordodes ferox* Camerano, 1897, a species previously described from the Democratic Republic of the Congo (Congo-Kinshasa) and an adjacent, not further specified region of the Republic of Congo (Congo-Brazzaville). Characteristic is the presence of enlarged and elevated simple areoles around the base of a thorn areole, in combination with further cuticular characters. This is the latest of a total of six species of horsehair worms reported from South Africa so far. Two species of praying mantids, *Polyspilota aeruginosa* (Goeze, 1778) and *Sphodromantis gastrica* Stål, 1858, have been identified as hosts of *Chordodes ferox*, while its distribution range in the region and the period of adult emergence from the host remain largely unknown.

## Introduction

A number of horsehair worms (Nematomorpha) are known and have been described from Africa, but most are occasional records, with few results from extensive sampling. Although still far from systematic sampling, Sciacchitano´s (1933, 1937, 1955, 1958, 1961) reports from the Democratic Republic of the Congo (then the “Belgian Congo”) are the most comprehensive records yet reported for an African country. For most other countries, only few species are known, which certainly underestimates the diversity of horsehair worms in most cases. From South Africa, six species have tentatively been reported thus far: *Chordodes capensis* Camerano, 1895 from the Cape of Good Hope; *Chordodes hawkeri* Camerano, 1902 from Grahamstown (erroneously reported as “Grahamstow”) in the Eastern Cape; *Chordodes ferox* Camerano, 1897 from Pietermaritzburg, KwaZulu-Natal; *Paragordius areolatus* Linstow, 1906 from Botshabelo, *Paragordius cinctus* Linstow, 1906 from the Mpumalanga Province; and *Beatogordius regularius* Heinze, 1934 probably from the Cape of Good Hope, as the location was only given in German as “Cap” (= Cape) (Camerano 1895, 1908, Linstow 1906, Heinze 1934).

Nematomorpha are long and slender worms, which develop parasitically in terrestrial insects (mainly praying mantids, carabid beetles, crickets and cockroaches) and a few other arthropods. Eventually, they emerge from these hosts into water for reproduction (see e.g. Hanelt et al. 2005, Schmidt-Rhaesa 2013). Early larval development takes place in water and larvae infect various aquatic animals as intermediate/paratenic hosts. This early phase of the life cycle and the transmission to the final host are not completely understood yet (see Hanelt et al. 2005, Schmidt-Rhaesa 2013 for reviews). About 360 species of nematomorphs are currently known, five from the marine environment (genus *Nectonema*) and the remaining (Gordiida) from freshwater.

Gordiids have comparably few diagnostic features. These are the shape of the posterior end and cuticular structures, such as bristles, spines and variously shaped elevations called areoles. Traditionally, cuticular samples were removed from the animals and investigated with the microscope, but now scanning electron microscopical (SEM) investigation has become the standard method, because with this technique characters can be documented at higher magnification and better quality. A number of African species have been reinvestigated using SEM (e.g. Schmidt-Rhaesa and De Villalobos 2002, Zanca et al. 2006a, b, De Villalobos et al. 2007, 2009) and recent species descriptions always include SEM analyses (e.g. Bolek et al. 2010, 2013, Hanelt et al. 2012).

We report here the determination of four specimens of horsehair worms, as *Chordodes ferox* Camerano, 1897 (at least the females). This species has been reported once before from Pietermaritzburg, KwaZulu-Natal, based on an old and poorly preserved specimen deposited in the Natural History Museum, London (see Schmidt-Rhaesa and Ehrmann 2001). However, the correctness of this identification remains questionable. More than 20 specimens of horsehair worms from different locations in Africa carry the label “*Chordodes ferox*” but it has not been established yet how many of these were adequately investigated and identified.

## Methods

**Figure 1.**
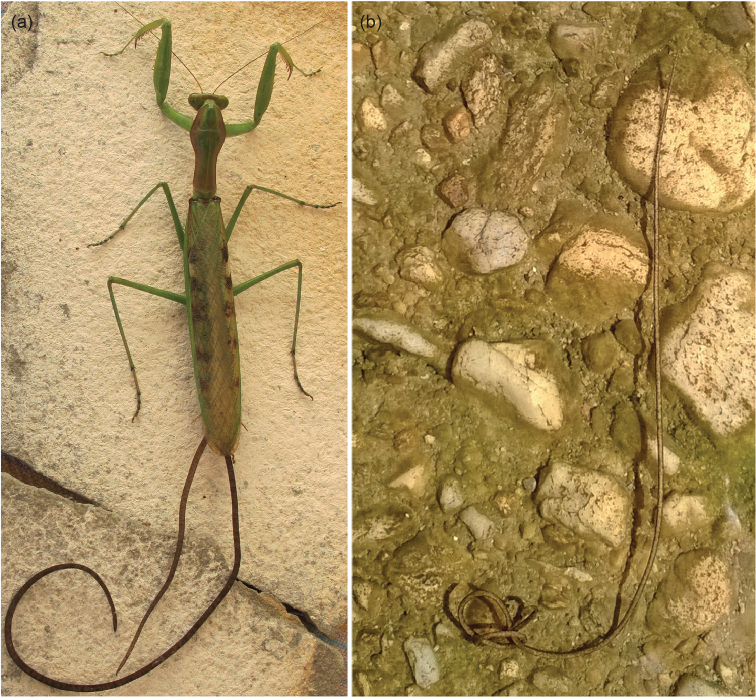
*Chordodes ferox*: **a** two specimens emerging from the posterior end of the praying mantid *Polyspilota aeruginosa* **b** free-living specimen on rocky substrata underwater showing a curled posterior end. Photos: Lynette Clennell, March 2015.

A total of four specimens were found in the Baviaanskloof Valley at Kudu Kaya Farm (river at campsite, 33°39'12.4"S, 24°34'59.7"E) in the Eastern Cape Province of South Africa, on 22 March 2015, while undertaking preliminary observations in the area. Two specimens emerged from the abdomen of a praying mantid, identified as *Polyspilota aeruginosa* (Figure 1a), attracted to the light at night. The other two specimens were lying free underwater (approximately 5–10 cm deep) on rocky substrata (Figure 1b), one in close proximity to an inactive, but still alive specimen of the praying mantid species *Sphodromantis gastrica*. Unfortunately, specimens were initially pooled together in the same container and, therefore, it cannot be established with certainty which of the specimens were the ones that emerged from the mantid *Polyspilota aeruginosa*. All specimens were taken alive to a laboratory for close observation under a dissecting microscope and later preserved in 99% ethanol.

Specimens are deposited in the Zoological Museum in the Centrum für Naturkunde of the University Hamburg under the numbers ZMH13363-ZMH13366.

For Scanning Electron Microscopy (SEM), both anterior and posterior ends of the body as well as a 2-3 mm long section from the middle region of the body were cut and dehydrated in an increasing ethanol:water gradient, then critically point dried, and coated with gold in a sputter coater. Observations were made using JSM-6360 (JEOL) SEM operating at 15 kV. Digital images were taken. Specimens comprise three females and one male, which are described separately here. All females are similar in their diagnostic characters, while the male differs slightly from the females in some characters. Figures used here come from the male (ZMH13363) and two females (ZMH13364 and ZMH13367), because in the third female (ZMH13366) the cuticle was dirty and less well preserved. For the description of cuticular structures (areoles) the terminology of Schmidt-Rhaesa et al. (2008) is used.

## Description

### Female

**Figure 2.**
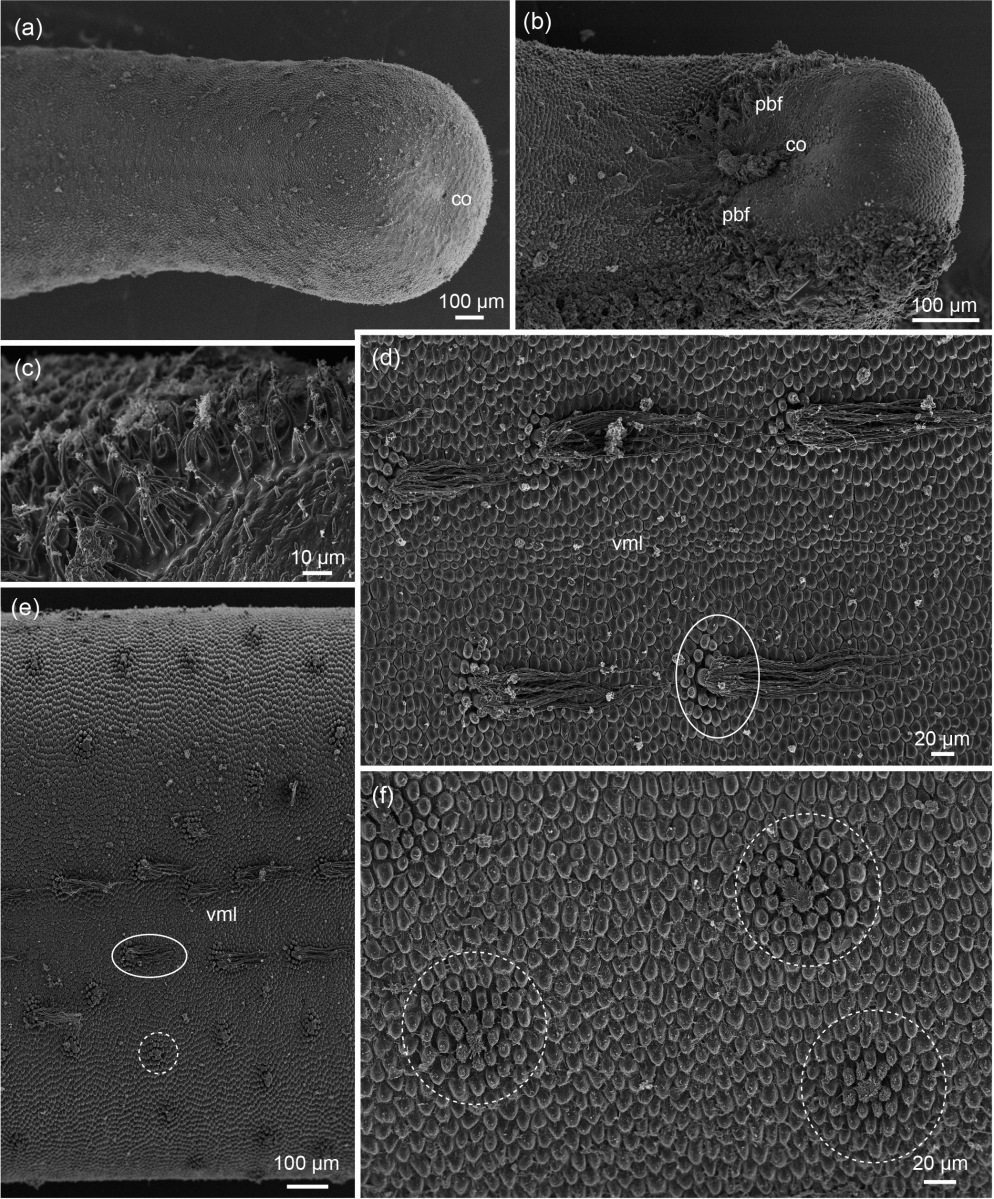
*Chordodes ferox*: **a** posterior end of female with terminal cloacal opening (co) **b** ventral view of posterior end of male with ventral cloacal opening (co) and precloacal bristlefields (pbf) **c** enlargement of bristles in the precloacal bristlefields **d** clusters of crowned areoles with long apical filaments (encircled) along both sides of the ventral midline (vml) in a female **e** overview of the ventral side of a female showing the distribution of clusters of crowned areoles with long (oval with unbroken line) and short (circle with broken line) apical filaments **f** clusters of crowned areoles in center and surrounding circum-cluster areoles (entire cluster encircled). SEM images: Andreas Schmidt-Rhaesa **a, f** from ZMH13366 **b, c** from ZMH13363 **d, e** from ZMH13364.

The posterior end of the females appears slightly swollen because the diameter of the body decreases slightly about 1 mm from the posterior tip (Figure 2a). The posterior surface is free of cuticular structures (areoles) and the cloacal opening is terminal (Figure 2a).

**Figure 3.**
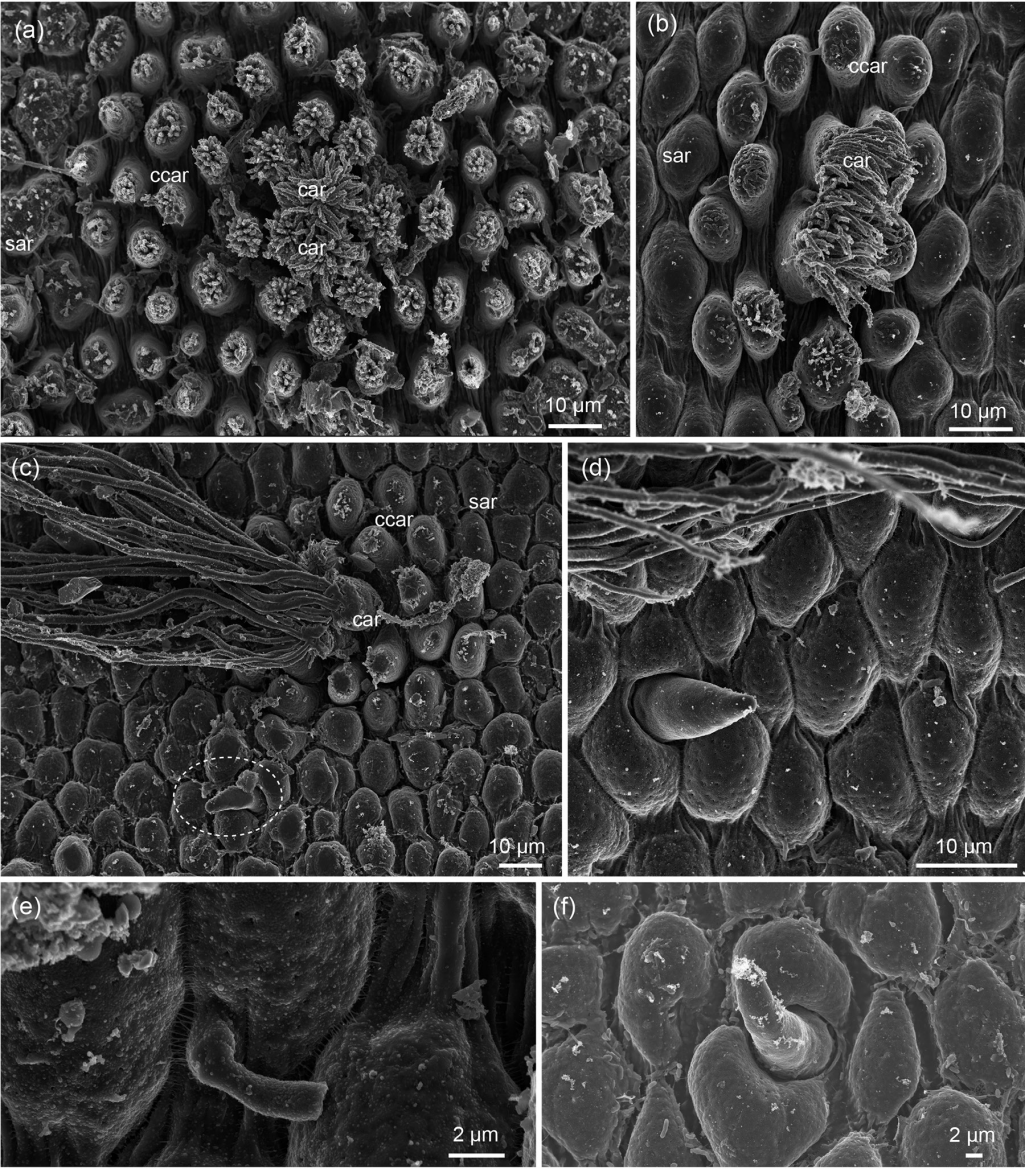
*Chordodes ferox*, higher magnification of cuticular characters: **a** cluster composed of two central crowned areoles (car) and surrounding circumcluster areoles (ccar) **b** cluster with fewer circumcluster areoles (Note transition between circumcluster areoles and simple areoles,sar) **c** cluster of crowned areoles with long apical filaments (encircled is a thorn areoles) **d, f** thorn areoles **e** isolated tubercle next to simple areoles. SEM images: Andreas Schmidt-Rhaesa **a** from ZMH13363 **b, d, e** from ZMH13364 **c, f** from ZMH13366.

Five different types of areoles were observed on the cuticle of the median piece. Simple areoles are roundish and have an almost smooth surface (Figures 2d, f, 3b-d). In the ventral midline they are slightly smaller than in other regions (Figure 2d). Crowned areoles occur in two forms, with long apical filaments along the ventral and dorsal midline and with short apical filaments in the lateral regions of the body (Figure 2e). Apical filaments of lateral crowned areoles are short to medium in length (about 10 µm), while the long filaments are up to 150 µm long. Crowned areoles occur in clusters with two central crowned areoles surrounded by circum-cluster areoles. These areoles have short bristles on top (Figure 3b). They decrease in size towards the periphery, which makes their number difficult to count. They are rarely few (ca. 14, Figure 3b), but usually between 18 and 20 (Figure 2f). Typical tubercle areoles were not observed, but rarely a tubercle areole with a strongly eccentric tubercle was found between simple areoles (Figure 3e). Few thorn areoles were found in the ventral region. Solid thorns are surrounded by 2-3 areoles, which appear sometimes (Figure 3f), but not always (Figure 3d) larger or more elevated than surrounding ones.

### Male

The posterior end exhibits the typical characteristics of the genus *Chordodes* (Figure 2b). The cloacal opening is ventral, about 250 µm from the posterior tip of the body. Its shape or the presence of circum-cloacal spines could not be observed due to a plug of dirt or sperm covering the opening. Antero-lateral of the cloacal opening are paired fields of spines (Figure 2c).

Simple areoles are roundish and their apical surface has small “knobs” (Figure 3a). Tubercle areoles were not seen, thorn areoles were found very rarely, while the surrounding simple areoles are not conspicuously larger than the others (not figured). Crowned areoles occur only with short apical filaments. Clusters of crowned and circum-cluster areoles are larger than in females and comprise > 40 circum-cluster areoles (Figure 3a).

## Discussion

Simple areoles, tubercle areoles, thorn areoles, crowned areoles in clusters surrounded by circum-cluster areoles and the presence of crowned areoles with conspicuously long apical filaments are typical characters found in *Chordodes* species (Schmidt-Rhaesa et al. 2008). The two other *Chordodes* species described from South Africa, *Chordodes capensis* Camerano, 1895 and *Chordodes hawkeri* Camerano, 1902 differ substantially from the specimens reported here. Both of these species have been reinvestigated recently using SEM techniques (Zanca et al. 2006b, De Villalobos et al. 2007). In *Chordodes capensis* both sexes are similar in their cuticular characters, with the exception that crowned areoles with long filaments are present in females, but absent in males (De Villalobos et al. 2007). The main characters of *Chordodes capensis* which differ from the specimens reported here are the presence of fine bristles on the surface of the simple areoles, the scattered presence of tubercle areoles and the absence of thorn areoles. In *Chordodes hawkeri*, both sexes are consistent in their cuticular characters, but the cuticle of the specimens analysed so far (lectotype male and paralectotype female) is in a relatively poor state and misinterpretations cannot be excluded (Zanca et al. 2006b). Crowned areoles with long filaments and thorn areoles were not reported in the specimens investigated by SEM. Other differences to the specimens reported here are the rough surface and irregular shape of simple areoles, isolated occurrence of crowned areoles (rather than in clusters), the scattered presence of tubercle areoles and the presence of another type of areoles, i.e. bulging areoles.

The characters of the female specimens investigated here correspond well with those reported in the description of *Chordodes ferox* Camerano, 1893. The species has been described based on one female specimen from the Republic of Congo (“French Congo”) found in an unidentified mantid species (Camerano 1893). According to Camerano (1893), *Gordius verrucosus* is, at least in part, a synonym for *Chordodes ferox*. This species has been described by Baird (1853) from a specimen of unknown locality. Subsequent authors have mentioned the location “Africa australis” (Diesing 1861, Villot 1874) or South Africa and Ceylon (Örley 1881), but while Diesing and Villot seem to relate to Baird´s specimen only, it is not clear whether Örley investigated further specimens. However, none of the descriptions was detailed enough or sufficiently well illustrated for a proper species description and the name was eventually regarded as “species inquirendae” (Camerano 1897).

The holotype of this species as well as further specimens have been investigated using SEM by De Villalobos et al. (2009). The described characters correspond to the characters described here. The combination of simple areoles with a smooth surface, crowned areoles with very short apical filaments, the presence of thorn areoles and tubercle areoles with an eccentric tubercle are regarded as characteristic for *Chordodes ferox*.

In addition, one potential further character of the specimens reported here is that the thorn areoles appear to be surrounded by a few large simple areoles. In most other species, thorn areoles are composed of a base, which resembles a “usual” areole and the thorn itself (compare, e.g. with figure 2C in Schmidt-Rhaesa et al. 2008). In the specimens described here, a basal structure is not recognized and instead the thorn is closely surrounded by simple areoles, which appear in some, but not all cases to be larger than the remaining ones. This arrangement has not yet been recognized as a character of taxonomic importance, but is, according to published figures, present in the species *Chordodes ferox*, *Chordodes maculatus* Sciacchitano, 1958 and *Chordodes madagascariensis* (Camerano, 1893). *Chordodes maculatus* is a species from the Democratic Republic of Congo (Sciacchitano 1958) and a SEM reinvestigation shows elevated simple areoles surrounding the base of thorn areoles (Figure 6B in De Villalobos et al. 2009). *Chordodes madagascariensis* is a species reported from Angola, the Democratic Republic of Congo, Guinea and Madagascar (see De Villalobos et al. 2009) and the shape of thorn and surrounding areoles can be seen in the original drawings (Figure 23 in Camerano 1897), but most clearly in the SEM reinvestigation (Figure 3D in De Villalobos et al. 2009). The main differences of these two species compared to *Chordodes ferox* are that *Chordodes madagascariensis* has high and slender crowned areoles (De Villalobos et al. 2009), while *Chordodes maculatus* has additional bulging areoles (De Villalobos et al. 2009). Therefore the cuticular characters of the female specimens reported here correspond best to *Chordodes ferox*, thus extending the distribution of this species to the southern African region.

The single male specimen available differs in its cuticular characters from the female specimens. The most conspicuous difference is the lack of crowned areoles with long apical filaments, which is a well-known sexually dimorphic character in the genus *Chordodes* (e.g. Schmidt-Rhaesa et al. 2008). However, as other differences concerning the surface of the simple areoles, the arrangement of areoles around thorn areoles and the number of circum-cluster areoles are also present, it is not clear whether this male, belongs to *Chordodes ferox*, despite having been found next to one of the females. All previous specimens reported to date are females, so it is not known how extensive sexually dimorphic characters are. There is no other species, which corresponds well to the characters of the male, therefore it cannot be assigned to any species currently known.

Praying mantids are the dominant host insects of horsehair worms of the genus *Chordodes* (Schmidt-Rhaesa and Ehrmann 2001). Both species recognized here, *Polyspilota aeruginosa* and *Sphodromantis gastrica*, have been recorded once before for worm specimens identified as “*Chordodes ferox*”, from Tanzania and from Pietermaritzburg, South Africa, respectively (Schmidt-Rhaesa and Ehrmann 2001). Both records were not from fresh specimens adequately documented in the literature, but from poorly preserved museum specimens. As the methods to investigate horsehair worms, as well as the awareness of diagnostic characters most suitable for their identification have changed and/or developed during the past decades, there is now a need for all old museum specimens to be re-investigated, at least those that have not yet been included in the peer-reviewed literature.

Although direct evidence of worm emergence was only observed from the abdomen of *Polyspilota aeruginosa*, the second mantid species, *Sphodromantis gastrica*, was found floating on the water in close proximity to one of the free-living worms, still alive but poorly active. There is little doubt, therefore, that it was involved in the release of the worm. Regarding timing and locality of the finding of the *Chordodes ferox* specimens, it is not known yet whether the period of emergence from the host is restricted to the austral autumn, as all specimens were collected towards the end of March. Searches during other seasons of the year are currently in progress but have so far yielded no further specimens. Regarding locality, all specimens were found in a relatively small riverine area within the Baviaanskloof valley. Although in a privately owned farm, the area is completely surrounded by the Baviaanskloof Wilderness Area, which is part of the Cape Floral Kingdom World Heritage Site. It is virtually certain that *Chordodes ferox* occurs through much of this area, particularly in the smaller water courses that act as tributaries to the main river.
